# Draft Genome Sequences of Two New Zealand *Xanthomonas campestris* pv. campestris Isolates, ICMP 4013 and ICMP 21080

**DOI:** 10.1128/genomeA.01247-15

**Published:** 2015-10-29

**Authors:** Dhairyasheel Desai, Jin-Hua Li, Eline van Zijll de Jong, Robert Braun, Andrew Pitman, Sandra Visnovsky, John Hampton, Mary Christey

**Affiliations:** a Bio-Protection Research Centre, Lincoln University, Canterbury, New Zealand; b New Zealand Institute for Plant and Food Research Ltd, Lincoln, New Zealand

## Abstract

*Xanthomonas campestris* pv. campestris is a necrotrophic bacterial pathogen of crucifers. We report here the draft genome sequences of isolates ICMP 4013 and ICMP 21080 from New Zealand. These sequences will facilitate the identification of race-specific factors in *X. campestris* pv. campestris.

## GENOME ANNOUNCEMENT

Black rot, caused by *Xanthomonas campestris* pv. campestris, is one of the most devastating diseases of cruciferous crops. In *X. campestris* pv. campestris, nine races have been described (1, 2) and the genome sequences are available for races 1, 3 and 9 (3–5). No sequences of isolates from races 2 and 4 have been published, despite race 4 being considered one of the most prevalent worldwide (6).

*X. campestris* pv. campestris is present on New Zealand crucifer crops. To identify the effectors and other virulence determinants associated with different isolates, the genome sequences of New Zealand isolates ICMP 4013 and ICMP 21080 were obtained by Illumina-based sequencing. Initially, pure cultures of each isolate were incubated overnight in LB media at 28°C. Genomic DNA was then isolated using the QIAamp DNA extraction kit (Qiagen). DNA samples with acceptable quality were then used to generate Illumina HiSeq2000 100-bp paired-end reads (Macrogen).

Approximately 5.6 Gbp and 6.1 Gbp of raw data were generated for ICMP 4013 and ICMP 21080, respectively. A total of 62,928,922 × 2 reads for ICMP 4013 and 69,035,858 × 2 reads for ICMP 21080 were subjected to quality control and trimming using Galaxy (7), and were then *de novo* assembled into contigs using a *k*-mer size of 75 in Velvet version 1.2.08 (8). The draft genome of ICMP 4013 is 4,972,211 bp in length, consisting of 80 contigs with an average 2479-fold coverage and an *N*_50_ of 29,472. The G+C content of the genome is 64.55 mol% and contains 4,428 predicted coding sequences (CDSs) and at least 60 tRNAs. The draft genome of ICMP 21080 is 4,927,810 bp, consisting of 412 contigs with a 2719-fold coverage and an *N*_50_ of 36489. The genome has a G+C content of 64.60 mol% and contains 4,371 predicted CDSs and 60 tRNAs.

Genes in both genomes were annotated by searching against the entire collection of FIGfams in the RAST (9) server. A function-based comparison of metabolic reconstruction (9) was then conducted to assess the differential functional roles in the genomes. The comparison revealed 12 functional roles differentially present in ICMP 4013 and eight in ICMP 21080.

The genomes of ICMP 4013 and ICMP 21080 were also compared to other *Xanthomonas* spp. by determining the “closest neighboring genomes” in RAST. Both genomes were closely related to that of the *X. campestris* pv. campestris type strain ATCC 33913. Further comparison of ICMP 4013 to ATCC 33913 revealed 61 functional roles differentially present in ATCC 33913 and 94 in ICMP 4013. The comparison of ICMP 21080 to ATCC 33913 revealed 70 functional roles differentially present in ATCC 33913 and 97 differentially present in ICMP 21080.

This is the first report of the genome sequencing of *X. campestris* pv. campestris isolates from New Zealand, which adds to the genomic data available and will consequently contribute to our knowledge of genomic diversity among isolates of *X. campestris* pv. campestris.

### Nucleotide sequence accession numbers. 

The genome sequences of *Xanthomonas campestris* pv. campestris ICMP 4013 and ICMP 21080 have been deposited in GenBank under accession numbers CP012146 and CP012145, respectively.
